# The impact of China's urbanization on ecosystem service value from the perspective of gross ecosystem product: a case study of Beijing-Tianjin-Hebei region

**DOI:** 10.1038/s41598-024-64655-8

**Published:** 2024-07-10

**Authors:** Yu Fan, Yun Zheng, Changgeng Jia, Youtao Song

**Affiliations:** 1 College of Environment, Liaoning University, Shenyang, China; 2 Institute for Finance Research, Shenyang, Liaoning China; 3 College of Marine and Environmental Sciences, Tianjin University of Science & Technology, Tianjin, China

**Keywords:** Beijing-tianjin-hebei urban agglomeration, Ecosystem services, Valuation, Gross ecosystem product, Climate-change policy, Environmental economics, Environmental impact, Sustainability, Ecology, Ecology, Environmental sciences, Environmental social sciences

## Abstract

Gross ecosystem product (GEP) is an aggregate measure of the monetary value of final ecosystem services, or the direct benefits that people derive from nature. GEP can provide decision makers with clear and competing evidence of the monetary value of ecosystem services. However, the relationship between GEP and urbanization has not been clarified which is not conducive to the decision-making role of GEP in the process of urban sustainable development. This work focused on the ‘Beijing-Tianjin-Hebei’ (BTH) urban agglomeration as a case study of the dynamics of ecological production amidst rapid economic and urban development, and coupled a spatial–temporal analysis of regional ecological change based on GIS (Geographic Information System) with economic valuation methods using official statistics and survey data. Results showed that from 2000 to 2020, the GEP increased from 1.55 trillion to 2.36 trillion, the value of provisioning services and cultural services increased from 0.51 to 0.71 trillion to 0.10–0.65 trillion. The value of regulation services showed an upward and downward trend (0.94–1.03–0.98) due to the rapid economic development in the Beijing-Tianjin-Hebei region. There were obvious spatial differences in the distribution of the GEP, in which Beijing, Tianjin, Tangshan, Cangzhou GEP accounted for 15%, 14%, 16% and 11%, respectively. During 2000–2020, there is a significant correlation between urbanization index (UI) and GEP in BTH, but the correlation between GEP and UI shows a trend of first increasing and then decreasing. The correlation between UI and EPS and ERS gradually decreases, and the impact of UI on ETS shows a significant positive correlation. In the future, it can be foreseen that urbanization will suppress the increase of GEP.

## Introduction

Ecosystem services (ESs) are the ecological features, functions, or processes that benefit people directly or indirectly^1,2^. Ecosystems and their processes not only make and keep the earth's life-support system going, but also they make the conditions that are necessary for human well-being. People and the biophysical environments in which they live make up complex "economic-social-natural" or "socio-ecological" systems, in which human actions change the conditions of the ecosystems that keep people healthy^3^.

The function of ecosystem services research in monitoring environmental change and managing resources is growing in importance. Ecosystems supply humans with provisioning services, regulating services, cultural services, and other supporting services. These service functions link ecosystems to human well-being and provide the foundation for the system's sustainable development and maintenance. Consequently, the value of ecosystem services has been assessed using a range of techniques prior to its incorporation into urban, landscape, and regional planning^4^.

Since the beginning of the twentieth century, cities have become the predominant human environment^5^. The rapid expansion of urbanization has intensified the strain on natural resources and ecological space, posing significant threats to the sustainable development of urban ecosystems and urbanization^6^

Although industrialization and urbanization enhance social, economic, and cultural development, it alters the structure and quality of ecosystems^7^, particularly land-use changes such as construction land expansion, deforestation, and wetland degradation^4^. This has a significant negative effect on ecosystem service value^8,9^. For the sustainable development of cities, therefore, a complete and systematic assessment of ecosystem services is essential. The assessment of ES in relation to urbanization is an effective tool to support decision-making processes for sustainable land use. This approach has important practical significance for coordinating regional sustainable development, optimizing land-use planning, and ensuring ecological security^10^.

There has been extensive research on the valuation of ecosystem services in the past. Costanza et al.^1^ pioneered the estimation of global ecosystem service value (ESV) in 1997, and the resulting research not only proposed a worldwide ecosystem-based service value assessment index system, but also quantitatively analyzed the global ecosystem service value^1^. Scholars in the MA (Millennium Ecosystem Assess) project have proposed that ecological conservation can be integrated into economic and social development decisions. In order to assess the value of such ecosystem contributions to human well-being, they have begun to implement this initiative by evaluating the global value of ecosystem services^11^. The United Nations Statistical Commission approved the "System of Environmental-Economic Accounting (SEEA) Core Framework" in 2012 and expected countries to apply the "SEEA Central Framework" similarly to the System of National Accounts. To complement SEEA-CF, the UNSC authorized SEEA-EA in 2021^12^.

In China, Ouyang et al.^13^ first introduced the concept of "gross ecosystem product" (GEP) as a parallel indicator to GDP in 2012. GEP refers to the entire value of goods and services provided by an ecosystem, of which there are seven types: forest, wetland, grassland, desert, and ocean^14^. GEP is an aggregate accounting indicator tries to give a thorough valuation of all relevant services that directly contribute to human wellbeing. It is the sum of the monetary value of final ecosystem services (without intermediate or "supporting" services to avoid double-counting)^14^. Numerous initiatives have been conducted in China to investigate GEP accounting across provinces, cities, and counties, and numerous local governments are testing it as a policy metric in pilot programs^15^. Jiang also used GEP to calculate the ecosystem service value of countries around the world in 2017 for the first time, and made recommendations for the sustainable development of the regional environment based on the results of regional GEP accounting and GDP^16^, Focusing GEP accounting on urban agglomerations can provide scientifically significant and administratively instructive insights into managing ecosystems for human wellbeing amidst rapid urbanization—a process that will continue in China for decades and likely add another 150 million city-dwellers within the next decade^17^.

Currently, many researchers have focused on the relationship between ecosystem services and their value and urbanization. The relationship between urbanization and ESs was explored in the Yangtze River economic Belt^18^, Taiwan Strait west coast urban agglomeration^19^, Shanghai-Hangzhou Bay Metropolitan Region^20^ Nanjing City^21^, and Pearl River Delta region^10^

Although the above studies have provided a key theoretical and empirical basis for exploring the interaction between urbanization and ESs, some questions remain to be addressed. Previous studies have explored the relationship between ES and urbanization through the benefit transfer approach (equivalent factor method) and the model method. Benefit transfer approach is easy for implementation and requires minimal data, it generally suffers from measurement and generalization errors, possibly leading to invalid and unreliable results^22^. The model method can only evaluate the physical quantity of ecosystem services, but not quantitatively evaluate the overall change of ES.

The GEP accounting focuses on the evaluation of ES and ESV through the functional value method, while taking into account the evaluation of multiple ecosystem service functions, and also quantitatively evaluating the overall ESV of the region through the value evaluation method, which is conducive to incorporating GEP accounting into the formulation of urban planning and development policies^23,24^.

At present, the relevant research on GEP accounting is only at the evaluation level, failing to clarify the internal driving factors of GEP changes, which is not conducive to the application of GEP in the management decision-making process^25–28^. Previous researchers had shown that urbanization is an important factor affecting ecological quality^20^. Therefore, it is necessary to clarify the relationship between GEP and urbanization, which is conducive to the sustainable development of future urban agglomerations.

This research focuses on the Chinese urban agglomeration of Beijing-Tianjin-Hebei (BTH): Beijing, Tianjin (Municipality), and Hebei Province. As a typical area of urbanization in China and China's economic and political center, the economy has flourished since reform and opening. The national "Twelfth Five-Year Plan" seeks to foster the integrated development of Beijing-Tianjin-Hebei and construct a world-class urban agglomeration and economic circle centered on Beijing. Rapid economic development, urban expansion, and population growth have put a tremendous strain on the ecological environment due to pollution. Beijing, Tianjin, and Hebei will begin a new phase of coordinated development in 2015, with ecological preservation and construction as one of the core development areas, and will strive to establish a comprehensive spatial pattern of coordinated development of the environment and economy by 2030. This research aims to investigate how rapid urban expansion affects ecological productivity (as measured by GEP) at the regional scale and how natural capital may be maintained sustainably under such conditions, which can provide a reference for other Chinese urbanized area’s evaluation of ecosystem services.

The research consists of the following: (1) The support of GIS technology and statistical data are combined to evaluate the value of GEP in the BTH region from 2000 to 2020 and land transfer changes; (2) Clarify the urbanization process in the BTH region and explore the relationship between GEP and urbanization; (3) Based on the GEP accounting results of BTH and the analysis of urbanization process, provide suggestions for sustainable development.

## Material and methods

### Study area

**Figure 1:**
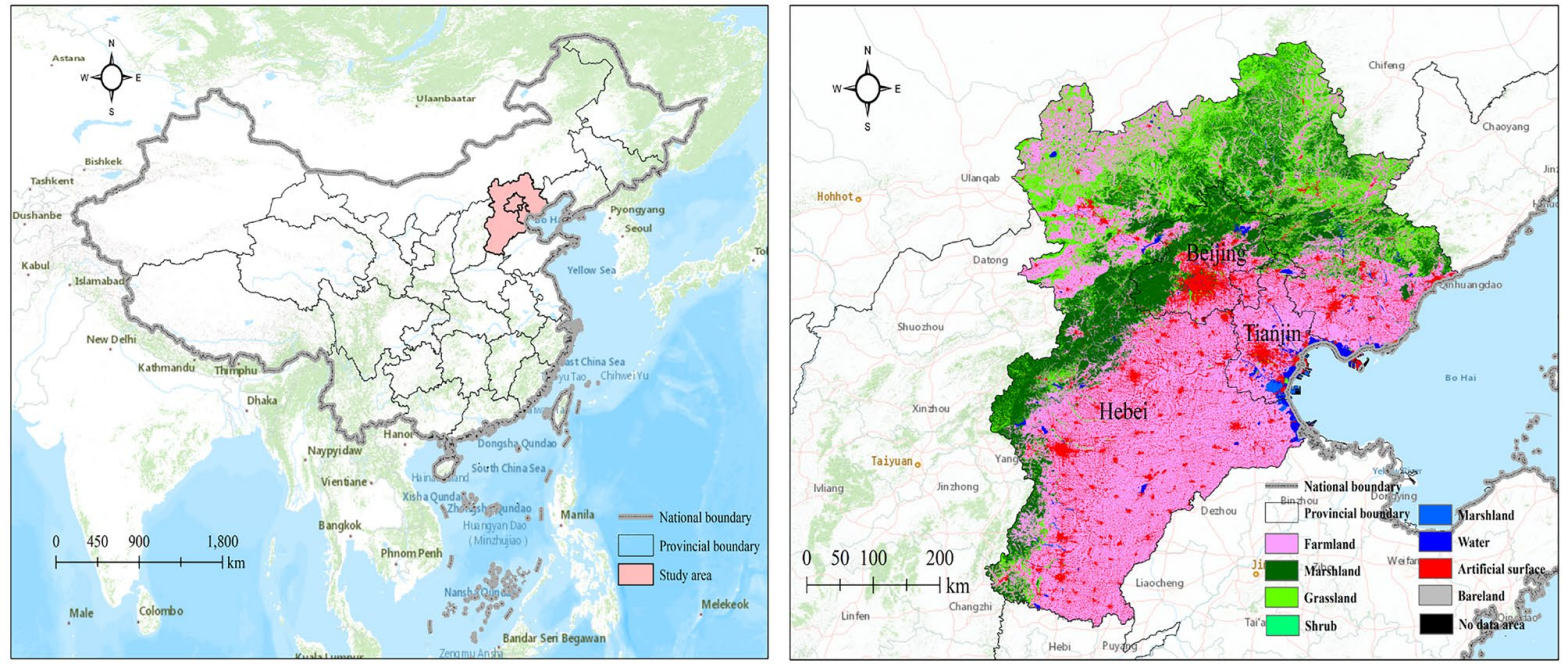
The location and ecosystem typology of the BTH urban agglomeration.

The BTH region (36010 N–42370 N, 113040 E–119530 E; see Fig. 1) constitutes China's capital economic circle. This region is located in the North China Plain, which connects the Inner Mongolia Plateau to the west with the Loess Plateau to the east. It is located in a warm, temperate temperature zone with a semi-humid continental monsoon climate. The annual sunshine is between 2500 and 2900 h, the annual total radiation is between 5000 and 5800 MJ/m^2^, and the annual average temperature is between 8 and 12.5 ℃. Precipitation is mainly concentrated in summer, accounting for 60–75% of the entire year’s amount. The overall area is approximately 2.30% of China at 21.68 km^2^. The BTH is northern China's most dynamic economic zone. This region's gross domestic product (GDP) was 845,808 billion RMB in 2019, accounting for 8.5% of the nation's GDP^29^. The growth of the BTH region is an important national strategic concern. It contributes significantly to the economic and social growth of China.

For the purposes of statistics and accounting, we classify the land use of the BTH ecosystem into six categories: woodland, grassland, cultivated land, wetland, and artificial surface. Forests comprise forests and shrubs, wetlands include rivers, lakes, natural wetlands and artificial wetlands, while others include barren terrain and underused land.

### Data acquisition

The classification of ecosystems was based on a 30 m-resolution land cover dataset derived from Chinese environmental disaster monitoring satellites (HJ-1A/B) and U.S. terrestrial satellites (Landsat OLI). To classify this remote sensing data into meaningful categories, an object-oriented multi-scale segmentation change detection method was applied. The sampling survey of remote sensing classification findings revealed that the accuracy of the first class was 95% and the accuracy of the second class was 89%^3^.

In order to calculate terrain factors and slopes, a digital elevation model (DEM) was used, which was obtained from the website https://www.gscloud.cn/ (30 m × 30 m). Soil data was collected from the Harmonized World Soil Database (HWSD), which can be accessed at http://www.fao.org/. Root depth data were acquired from the National Earth System Science Data Center (http://www.geodata.cn/) (1 km). The datasets of MODIS13Q1 NDVI generated every 16 days at 250 m spatial resolution were downloaded from the NASA Earthdata Center (https://earthdata.nasa.gov/); The meteorological station data for the study area were sourced from the China Meteorological Data Service Centre (https://data.cma.cn/). To transform the point data into raster data, the monthly precipitation, temperature, and sunshine percentage data from 2000 to 2020 were interpolated using the thin-plate splines method (30 m). Annual potential evapotranspiration was obtained from National Qinghai Tibet Plateau Scientific Data Center (https://www.tpdc.ac.cn) (1 km).

Socioeconomic, population, pollution monitoring data were obtained from publicly accessible official statistics maintained by relevant provincial and national government departments, such as the "China Urban Statistical Yearbook (2000–2019)", "Hebei Economic Statistical Yearbook (2000–2019)", "Beijing Statistical Yearbook (2000–2019)," and "Tianjin Statistical Yearbook (2000–2019)". In addition, information on the biophysical and economic dimensions of ecosystem services was gathered from official data sources and pertinent literature.

### GEP accounting method

**Figure 2:**
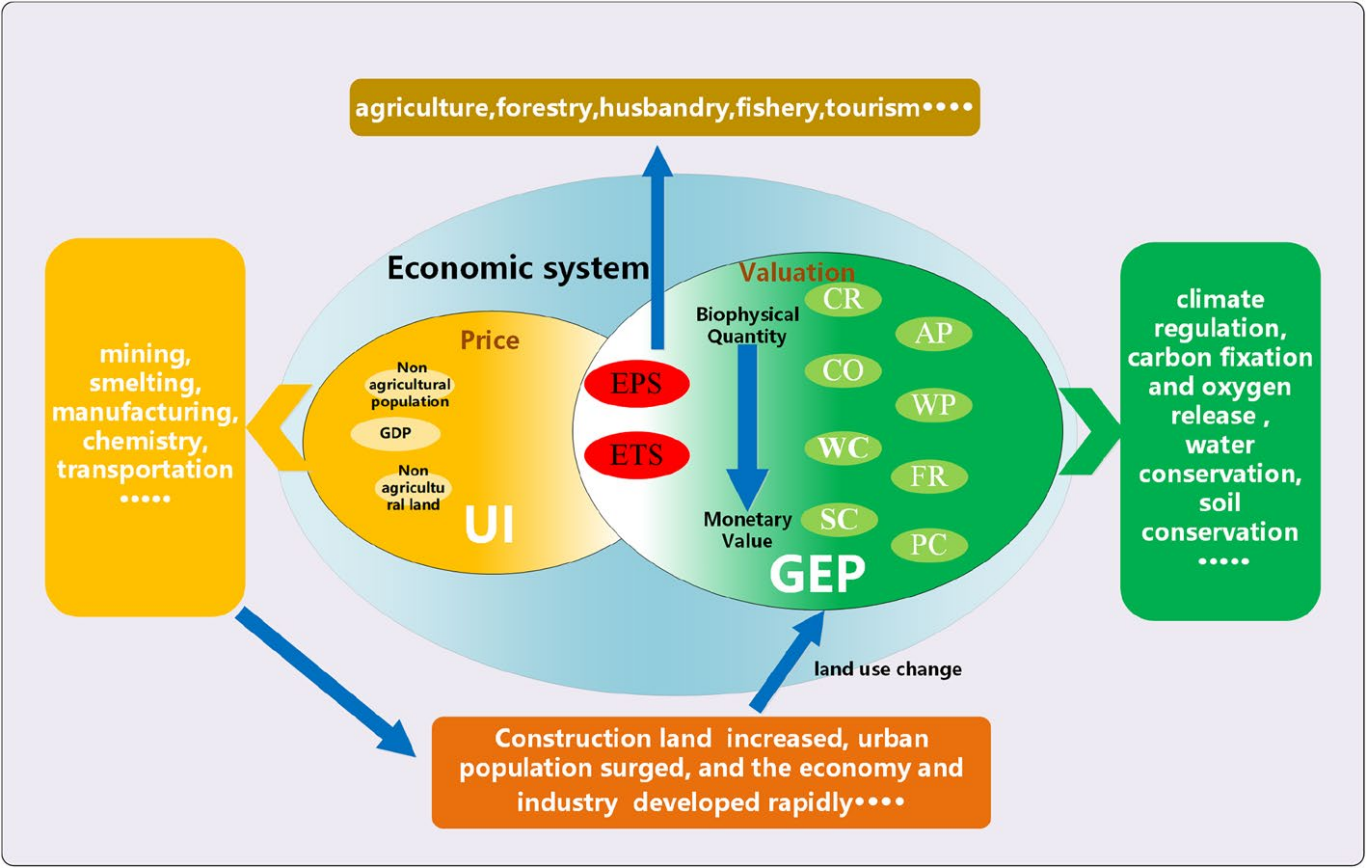
The framework used to measure GEP.

Measuring ecological services could facilitate the evaluation and analysis of their economic value to human welfare. After reviewing previous research conducted by^1,2,16,30^, the MA^31^, we developed a framework for measuring the GEP of BTH which is shown in Fig. 2.

The GEP accounting system of BTH is built in Eqs. (1, 2). The system of accounting indexes for the GEP in the BTH consists of three major categories of services: provisioning services, regulating services, and cultural services. Provisioning services mainly include agricultural products, forest products, livestock products, fishery products, and freshwater resources; regulating services mainly include water conservation, soil conservation, flood storage, carbon sequestration, oxygen supply, air purification, water purification and climate regulation, pest control functions; cultural services mainly include leisure and tourism values.

1 $$GEP = EPS + ERS + ETS$$

2 $$ERS = CR + CO + WC + SC + AP + FR + PC$$

ERS is the value of the ecosystem regulating services. EPS is the value of the ecosystem provisioning service, and ETS is the value of the ecosystem tourism services^32^. CR is the value of the climate regulation service, CO is the value of the carbon fixation and oxygen release service, WC is the value of the water conservation service, and SC is the value of the soil conservation service, AP is the value of the air purification, WP is the value of the water purification, FR is the value of the flood regulation, PC is the value of the pest control.

The GEP is calculated by using biophysical variables and pricing of several ecosystem services. In particular, the value of physical services is mostly determined by the direct market price technique, the value of regulating services is primarily determined by the alternative cost method, and the value of non-physical services is determined by the tourism cost approach. Section S1 provides more detailed evaluation indexes and data descriptions.

### Selection of urbanization index (UI)

Urbanization is a broad idea and a methodical process. It is commonly considered that the urbanization process focuses primarily on four factors: economic level, population increase, urban spatial expansion, and cultural communication^33^. combined with prior research^20,34^. Urbanization is a process of transformation from rural regional system to urban regional system. The urbanization rate refers to the proportion of urban resident population to the total resident population^35^. We described the urbanization levels of the BTH in terms of population growth, economic development, and construction land expansion. This study employs three indices to quantify UI, including non agricultural population proportion *(NAP)*, *GDP* (yuan/km^2^), non agricultural land proportion (*NAL*).

Calculate the urbanization index (UI) using the formula given below as Eq. (3). Individual index was range-standardized to a value between 0 and 1 and then averaged to determine the UI value^36^. The urbanization index formula is as follows:

3 $$UI = \frac{1}{3} \times \left( {NAP + GDP + NAL} \right)$$

where *UI* refers to the urbanization index; *NAP* is the standardized non agricultural population proportion; *GDP* is the standardized *GDP* density; *NAL* is the standardized non agricultural land proportion.

The GEP of each city was extracted using the Sample tool. Pearson correlation analysis and significance tests were employed to identify the interactions between GEP and UI.

Significant negative correlations (p < 0.05) denote significant trade-offs, while significant positive correlations (p < 0.05) represent significant synergy relationships between pairs of ESs^37^.

## Results

### Ecosystem structure and change

**Figure 3:**
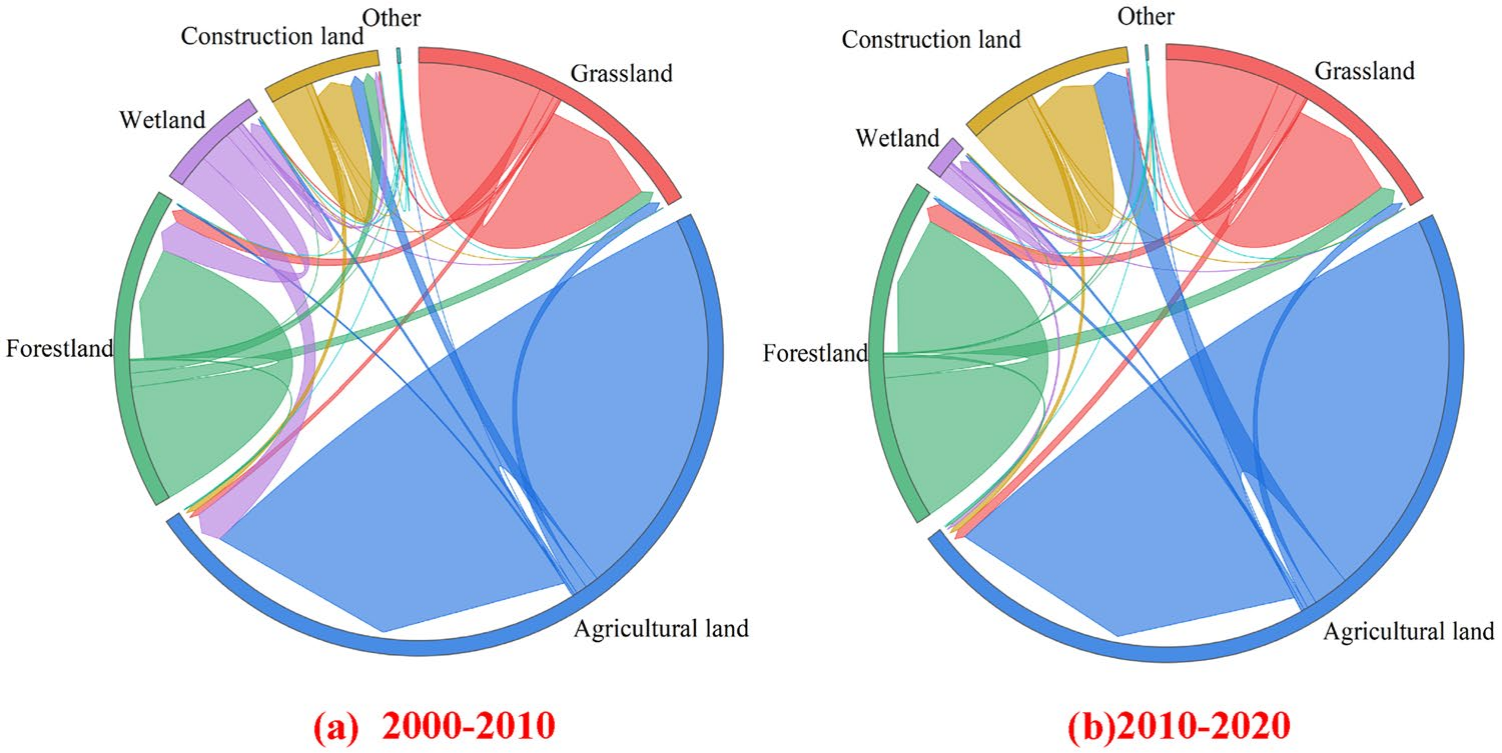
Land use change in BTH region.

There were diverse ecosystems exist in the BTH region (Fig. 3). From 2000 to 2020, the BTH region has the highest proportion of cultivated land, including around 50% of BTH land area, primarily distributed in the southeast. Forestland and grassland contribute for around 17 to 20% of the total land area and are primarily located in the northwest. Approximately 8 to 12% is used for construction, primarily in metropolitan areas. Wetlands account for the lowest percentage, roughly 2%, and are primarily found in coastal regions.

We employ GIS remote sensing technologies to examine the change in land use in the BTH from 2000 to 2010 and 2010 to 2020, which were shown in Fig. 3. Due to the rapid growth of the BTH economy between 2000 and 2020, many types of land in the BTH region saw significant changes. From 2000 to 2010, the area of construction area expanded by approximately 20% due to the conversion of forest land and agricultural land, while the area of forest increased by 20% due to the conversion of agricultural land and grassland. The area of wetland rose little, while grassland and arable land remained relatively unchanged.

In the second stage, the intensity of land use change was diminished compared to the first. The area of development land expanded by around 50%, primarily at the expense of cropland. As a result of the conversion of farmland to construction area, forest, grassland, etc., the amount of cropland has decreased by 7%.

### GEP values and changes

**Figure 4:**
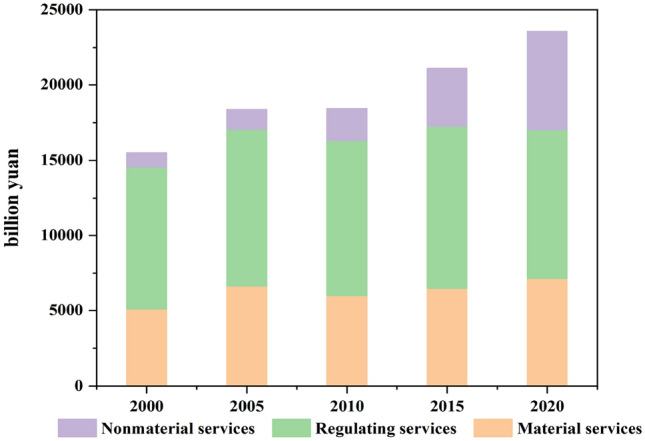
2000–2020 GEP changes of BTH urban agglomerations.

Figure 4 illustrates the change of GEP in the BTH from 2000 to 2020. In 2000, 2005, 2010, 2015, and 2020 (at current prices), the GEP was 1.55, 1.83, 1.84, 2.11, and 2.35 trillion yuan, with a consistent upward trend. In each year of the GEP composition, the value of regulating services accounted for between 40 and 60%, the value of provisioning services accounted for approximately 30%, and the value of cultural services accounted for the least, with a maximum of no more than 28%.

To further investigate the evolution of GEP in the BTH region, we calculated the GEP of several cities and municipalities in the BTH region from 2000 to 2020 (Table 1). Table 2 displays the evolution of each city's GEP from 2000 to 2020, revealing a tendency of steady growth followed by stability. From 2000 to 2010, the cities with the highest GEP were Beijing, Tianjin, Shijiazhuang, Tangshan, and Cangzhou, each with a GEP greater than 100 billion yuan, and in Beijing, Tianjin, and Tangshan, the GEP at each stage was greater than 200 billion yuan. Cities having a GEP of less than 100 billion yuan include Qinhuangdao, Handan, Xingtai, Baoding, Chengde, and Langfang. For PGEP (GEP/km^2^), the cities Tangshan, Beijing, Tianjin, and Cangzhou are included, and the PGEP at each level exceeds 10 million yuan per square kilometer. Shijiazhuang, Qinhuangdao, Handan, Baoding, Zhangjiakou, etc. are cities with low PGEP.

**Table 1:** GEP (billion yuan and PESV (million yuan/km^2^) in BTHUA from 2000 to 2020.

Area	2000		2005		2010		2015		2020	
	GEP	PGEP	GEP	PGEP	GEP	PGEP	GEP	PGEP	GEP	PGEP
Beijing	2369	1444	2397	1461	2819	1718	3141	1914	2703	1647
Tianjin	2135	1787	2394	2004	2521	2110	3063	2564	3086	2583
Shijiazhuang	1005	695	1023	707	1178	814	2530	1749	1883	1302
Tangshan	2457	1824	2975	2209	3033	2251	3150	2338	3353	2489
Qinhuangdao	591	756	692	886	835	1069	1036	1326	1239	1587
Handan	542	452	413	344	494	412	602	502	1323	1103
Xingtai	832	671	746	602	916	739	790	637	996	804
Baoding	636	279	707	310	950	416	1099	482	1926	844
Zhangjiakou	1372	373	1385	376	1224	333	1426	387	1659	451
Chengde	837	212	926	235	870	220	1146	290	1816	459
Cangzhou	1678	1250	2335	1740	2192	1634	1736	1294	2261	1685
Langfang	419	652	1034	1608	511	795	566	881	690	1074
Hengshui	648	735	1346	1527	887	1007	840	953	651	739

**Table 2:** The urbanization index of BTH.

Levels	2000	2005	2010	2015	2020	2000–2020 (%)
Non agricultural population proportion (NAP)	0.30	0.35	0.42	0.47	0.55	83
GDP (yuan/km^2^)	344.44	728.82	1306.84	3193.92	3924.84	1041
Non agricultural land proportion (NAL)	0.37	0.41	0.39	0.46	0.54	46

**Figure 5:**
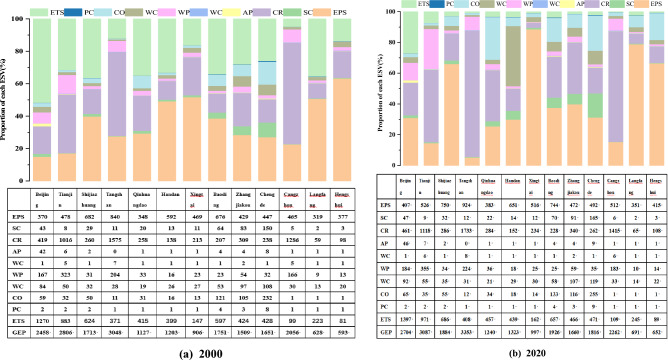
Proportion of the value of each ecosystem service function to ESV of the BTH in 2000 (**a**), and 2020 (**b**).

Figure 5 shows the proportion of the total ecosystem services of 13 cities in ESV in 2000 and 2018, respectively. From 2000 to 2012, the proportion of GEP varied slightly across cities.

In 2000, high GEP cities such as Tianjin, Shijiazhuang, and Cangzhou accounted for more than 40% of regulation services, with climate regulating and water conservation accounting for the highest value, while Beijing and Shijiazhuang GEP accounted for more than 40% of the value of cultural services and provisioning services. The regulation services value accounted for more than 50% in each city. In cities with low GHG emissions, like as Qinhuangdao, Chengde, Handan, and others, the total value of provisioning services and cultural services exceeds 50% and plays a leadership role. In terms of regulating services, climate regulation, carbon sequestration, and oxygen release value play a major role.

The fraction of regulating service value in cities with a high GEP will increase further by 2020. In Cangzhou, Tangshan, and Tianjin, for instance, the value of climate adaptation accounts for more than 60% of GEP, and the value of climate adaptation and water conservation play a leading role. The proportion of regulating service value in cities with low GHG emissions has also increased since 2000. Compared to 2000, the proportion of regulating service value in Zhangjiakou City, Chengde, has climbed by more than 30%. Leading roles are played by the functions of soil and water conservation, climate adaptation, and water conservation.

### Impacts of urbanization on GEP of BHT

In 2020, the population density was 343.61 inhabitants per square kilometer, while in 2019 it was 475.55 inhabitants per square kilometer. In 2000, the GDP density was 344.44 yuan/km^2^ and in 2019, it was 3894.84 yuan/km^2^. The percentage of development land increased from 0.03 in 2000 to 0.12 in 2019. They grew by 38%, 1030%, and 300%, respectively. The expansion of GDP density was considerably more rapid than that of the others (Table 2).

The urbanization index of BTH is shown in Table 2, The BTH urbanization index increased significantly during 2000–2020, which the proportion of non-agricultural population increased by 83% from 0.3 in 2000 to 0.55 in 2020. The population density increased from 344 yuan/km^2^ for 2000 men to 3924 yuan/km^2^ in 2020, for an increase of 1041%. The proportion of non-agricultural land increased by 46% from 0.37 in 2000 to 0.54 in 2020.

**Figure 6:**
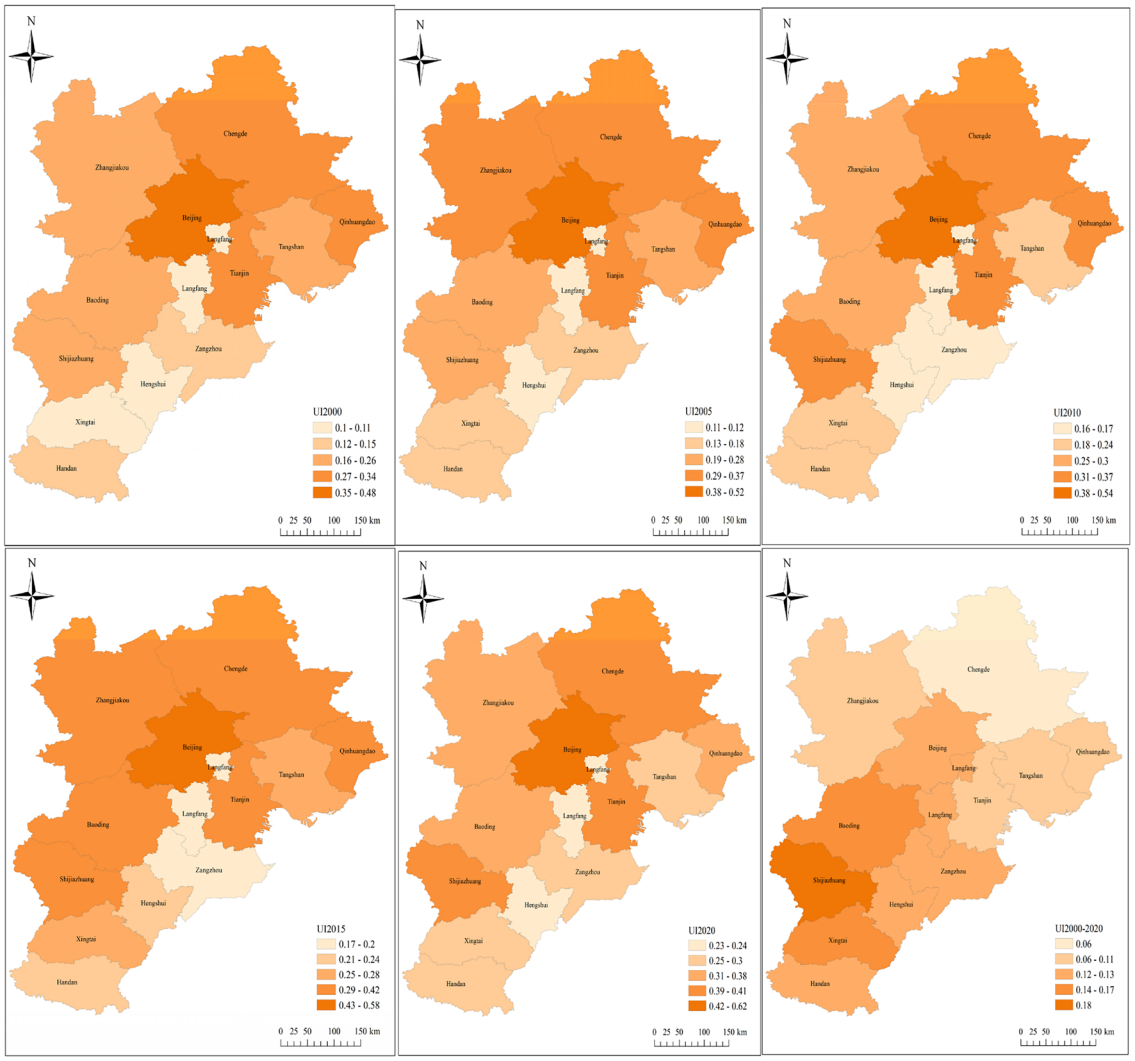
Comprehensive urbanization level of the BTH in 2000–2019.

Figure 6 illustrates the regional and temporal patterns and dynamic properties of urbanization indicators for the BTH region between 2000 and 2020. The spatial patterns of NAP, GD, and NAL were relatively stable from 2000 to 2020. The highest levels of urbanization were mostly focused in two megacities: Beijing and Tianjin. The temporal patterns of the three indicators were essentially same, with rising averages throughout the BTH region.

Comprehensive evaluation of the urbanization level of BTH based on three indicators: economy, population, and land (Fig. 6). From 2000 to 2020, the differences in the urbanization levels of thirteen cities in the BTH region were determined. From 2000 to 2020, each city's urbanization index demonstrated an increased trend. In comparison to 2000, the urbanization index of Beijing, Tianjin, Shijiazhuang, etc. rose by more over 50%. By 2020, the following will be the urbanization index rankings for thirteen cities: Beijing > Tianjin > Shijiazhuang > Chengde > Qinhuangdao > Baoing > Zhangjiakou > Tangshan > Handan > Xingtai > Cangzhou > Hengshui > Langfang, respectively 0.61, 0.41, 0.40, 0.39, 0.38, 0.37, 0.35, 0.30, 0.28, 0.27, 0.26, 0.24, 0.23.

**Figure 7:**
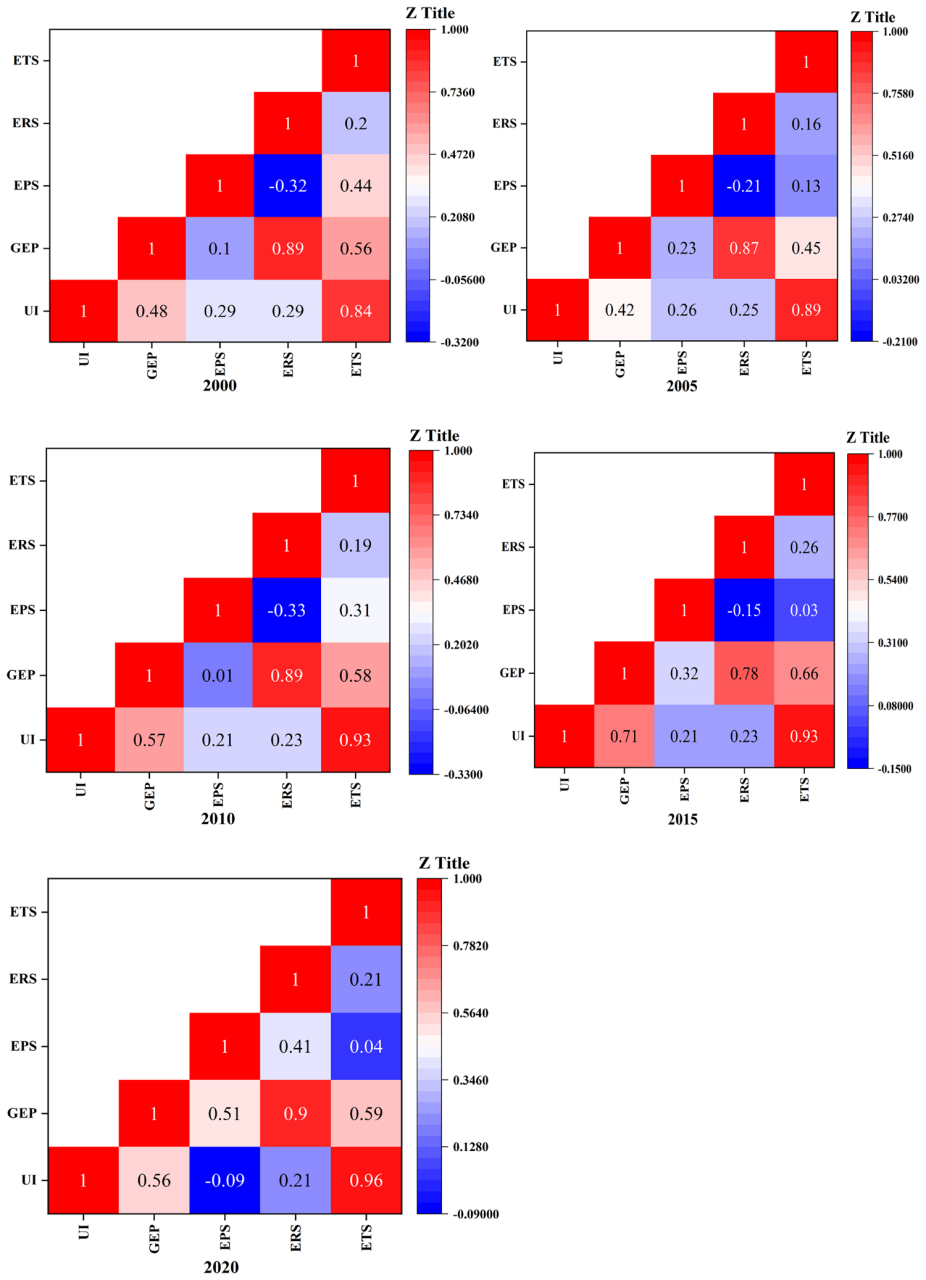
The correlation of UI and GEP in BTH from 2000 to 2020.

This study examines the relationship between the urbanization index and GEP by Pearson correlation analysis^32^. The relationship between GEP and UL in BTH is shown in Fig. 7. During 1990 and 2020, the UI of BTH was significantly correlated with GEP at a p < 0.001 level. UI is positively correlated with GEP, but the correlation between GEP and UI shows a trend of first increasing and then decreasing, from 0.48 in 2000 to 0.70 in 2015, and then decreasing to 0.55. The correlation between UI and EPS gradually decreased, from 0.28 in 2000 to 0.21 in 2010. However, after 2015, UI and EPS showed a negative correlation, and this negative correlation continues to increase. The correlation coefficient increased from -0.09 in 2015 to -0.17. The correlation between UI and ERS is similar with EPS, with the correlation coefficient decreasing from 0.28 in 2000 to 0.21 in 2020. The impact of UI on ETS shows a significant positive correlation, which increased from 0.83 in 2000 to 0.96 in 2020.

## Discussion

### Land use and GEP change of BTH

GEP accounting is now being piloted in numerous locations of China, particularly in places with significant ecological function (such as Qinghai Province). The GEP framework can properly measure the advantages of their ecosystems to humans, although GEP practices are uncommon in congested urban environments. In the Beijing-Tianjin-Hebei (BTH) region of China, for instance, the urban population and natural ecosystems are intimately integrated, and ecosystem services have a greater direct impact on human existence. China has achieved great progress in industrialization and urbanization since 1978, when reform and opening up began^38^. During this time period, the BTH region's ecology has seen significant changes, which is consistent with the region's rapid population and economic growth and the extension of urban land. Similarly, similar urbanization processes have a substantial effect on the quantity and quality of ecosystems.

National development strategies and China's entrance to the World Trade Organization (WTO) in 2001 contribute significantly to this phenomenon in the context of economic globalization^3^. This has a significant impact on urbanization in China^39^. As one of the most important urban agglomerations in China, BTH should prioritize quality over speed and be devoted to the development of high-quality urbanization, rather than pursuing the rapid rise of urban population and urban area expansion in the future. Urban construction should increase land integration, rather than occupying arable land or natural ecological systems, and promote healthy ecological development^40^. The rapid development of BTH from 2000 to 2019 led to the rapid increase of construction land and the sharp loss of cropland, forest, and wetlands (Fig. 4), especially cropland, which accounted for more than 50% of the newly added construction land, which poses a serious threat to regional food security and ecological protection^41^, this is consistent with Guo et al.^40^ findings.

For ESV evaluation, we employ the GEP accounting paradigm to investigate the effect of land transfer and urbanization on ESV. Consistent with the findings of Zou et al.^3^. The GEP in the Beijing-Tianjin-Hebei region exhibited an increased trend from 2000 to 2020, as shown by the data^3^. During this time, both the value and share of non-regulated services in GEP expanded dramatically. In 2000, non-regulated services accounted for 40% of the value of services. By the year 2019, the value of unregulated services will account for 58% of the total. This was primarily due to the following: economic development brought about by rapid urbanization improved the living standards of local residents as well as visitors from other parts of the country, and the demand for recreational tourism increased accordingly^42^, during the process of rapid urbanization, the addition and upgrading of infrastructure made tourism much more accessible, and the corresponding growth of regional traffic led to a substantial increase in the number of tourists. This raises both the GDP and GEP.

From 2000 to 2020, both the value and proportion of regulating services clearly decline. Comparing 2020 to 2000, the value of regulating services declined by 20%. The ecosystem regulating service function is essential for human life and the ecosystem itself, delivering many benefits to regional and even global humans and ecosystems (such as water conservation, carbon sequestration and oxygen release, and climate regulation). Due to changes in land transfer, the value of regulating services in the BTH area grew between 2000 and 2020 before declining. Between 2000 and 2020, marsh and woodland ecosystems expanded dramatically, while forest cover increased by 10%. This is primarily owing to China's "returning cropland to forest" program, which has resulted in the conversion of agricultural and grassland land. China converted a total of 403 million of cropland to forest from 1999 to 2008. The degradation of wetlands and cultivated land (reduced by 17% and 7%, respectively, from ten to nineteen years) is the primary cause of the decline in the value of regulation services, with wetlands providing crucial functions such as climate regulation, flood regulation, and storage.

This study is the first to assess the value of ecosystem services in the BTH region through GEP, but this is just an attempt. In previous similar studies, some scholars have paid attention to the supply capacity of the ecosystem in the BTH region^34,43,44^. It includes food supply, water supply, soil erosion, carbon dioxide fixation and oxygen release, but neglects ecosystem services such as cultural services. BTH has 522 3A-level scenic spots, attracting more than 1 billion domestic and foreign tourists in 2020 (Statistical Yearbook), and the economic benefits brought by ecological natural landscapes are considerable. Hu et al.^45^ and Guo et al.^40^ evaluated the value of ecosystem services in the BTH region based on the equivalent factor method, which can evaluate the value of multiple ecosystem services. However, this method of ignores regional development differences. GEP can provide decision makers with clear and compelling evidence of the monetary value of ecosystem services^14^. GEP can contribute to achieving important societal objectives, such as sustainable development, by bringing the value of ecosystem services and trends in ecosystem assets into public and private sector decision making and investment planning.

### Urbanization and GEP

Calculating the value of ecosystem services is a burgeoning area of study with significant practical implications. This strategy can illustrate the differences in value of ecosystems of the same type in various places. GEP accounting is still in its infancy, but it has absorbed previous research experience in accounting methods (such as the Millennium Ecosystem Assessment and the United Nations System of Environmental Economic Accounts).

We link GEP with urbanization development for the first time, explore the relationship between the rapid development of typical Chinese cities and ecosystem services, and provide a basis for the sustainable development level of dense urban areas in China. Our results found that urbanization in BTH area has a positive correlation with GEP, EPS, ERS, but this correlation is decreasing, especially EPS, the impact of urbanization on EPS from 8.0 to − 0.17, which shows that the relationship in the future, population, tourism, economic rapid development in urbanization, which promoted the EPS and ETS increased significantly, and the development of urbanization may still not exceed the upper limit of the ecosystem, so the UI has the least impact on ERS. This is consistent with the findings of Zou et al.^3^. We will focus on the interaction between GEP and urbanization in future work.

### GEP works in parallel with GDP

The Chinese government now mandates the inclusion of ecological advantages in the evaluation of local government performance. In addition to using GDP to evaluate economic progress, use GEP as a benchmark for administrative and development performance. Implementing the dual evaluation system of GEP and GDP allows for an accurate evaluation of the relationship between environmental protection and economic growth. Many local governments in China have chosen GEP as an exploratory policy on the basis of this premise^46^.

GEP increases social-economic-natural evaluation by measuring nature's contribution to humanity. Assessing GEP and GDP in simultaneously promotes integrated economic and environmental improvement^16^.

**Figure 8:**
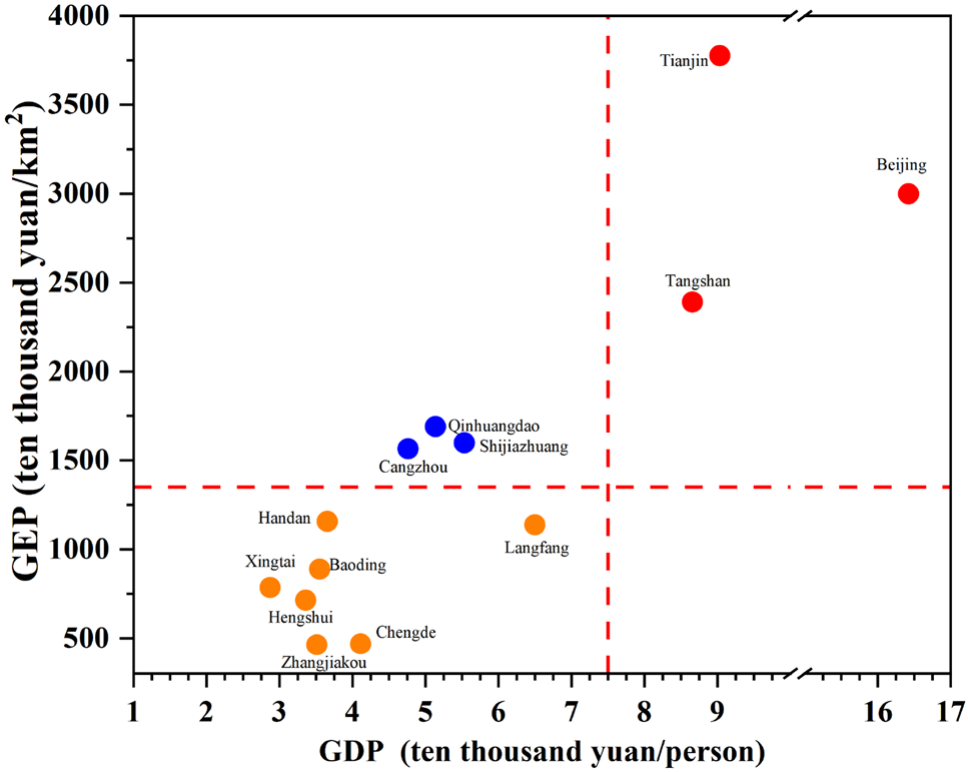
Classification of 13 cities on the basis of GEP and GDP. Group I, located in the upper-right-hand quadrant, GDP. Group II, located in the upper-left-hand quadrant, Group III, located in the lower-left-hand quadrant,. Group IV, located in the lower-right-hand quadrant.

We've also analyzed the GEP/GDP ratio per country. GEP per unit area is the ordinate while GDP per capita is the abscissa. The 13 cities are separated by their GEP (2020) and GDP values (2020). Group I has high GEP and GDP, Group II has high GEP but low GDP, Group III has low GEP but high GDP, and Group IV has low GEP and GDP (Fig. 8).

Group I, which includes cities such as Beijing and Tianjin, has a high GEP and GDP, indicating that cities in this quadrant are currently in a mode of sustainable development with a good ecological and environmental protection status and economic growth.

Group III cities, including Qinhaungdao and Cangzhou, have a lower GDP and GEP, indicating that they must increase not only ecological and environmental protection measures but also economic development.

Three cities, Handan, Xingtai, and Langfang, are members of Group IV, which has a high GEP and a low GDP. These cities should take into account the connection between their ecological environment and economic development, and translate green ecological values into economic values in a sustainable manner.

Currently, Chinese academics are attempting to employ ecological goods as the fourth industry; we use the operational ecological product index and the public ecological product index to examine the eco-products fourth industry structure of the BTH region at the city level.

The public ecological product index is the ratio of public ecological product value to GEP. The operational ecological product index is the ratio of operational ecological products to GEP. Operational ecological goods relate to the fully commercialized portions of EPS and ETS.

**Figure 9:**
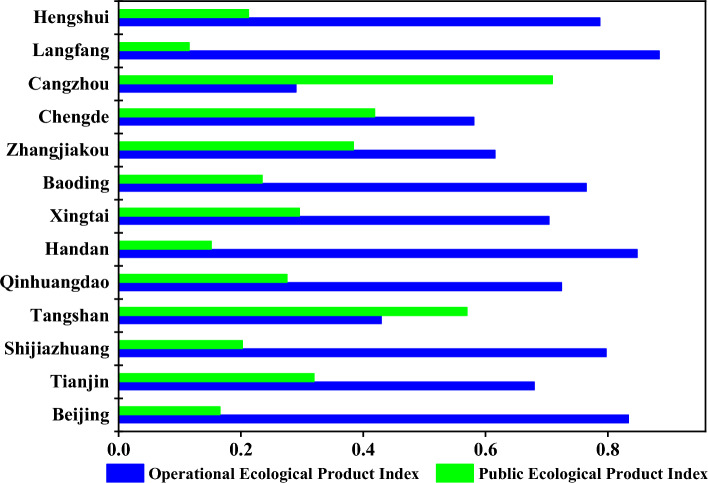
Analysis of eco-products fourth industry structure index in BTH.

Figure 9 depicts the 2020 fourth industrial structure index for the BTH region. The BTH region is a dense urban area in China, and the business ecological product index of most cities is greater than the public ecological product index, indicating that most cities in the BTH region have a relatively high degree of marketization of ecosystem service value. Only the public ecological product index of Tangshan and Cangzhou is greater than the commercial ecological product index, indicating that the ecological function of the region is exceptional.

### Limitation

The GEP accounting system comprehensively considers the benefits brought by various types of ecosystems to human beings which based on land data, statistical and meteorological stations data of the study area to calculate the value of ecosystem services, such as product supply value, mainly from regional statistical yearbook data. However, the GEP accounting system still has many deficiencies. First, the value of material and immaterial services must be included in the complete value of regional ecological production that GEP seeks to capture. Due to data limitations, however, the value of material services is estimated using official statistical reports on agricultural, forestry, animal husbandry, and fishery products, whereas the value of cultural services is reflected in the value generated by ecotourism, and cultural services are evaluated using regional tourism income and population. value. It is difficult to distinguish between the contribution of natural ecological processes and the added value of artificial inputs (such as fertilizers and machinery) in this process^3^. The calculation process of some indicators does not consider the relationship between supply and demand, which may lead to high calculation results. Perception of ecosystem services is often linked to the use and non-use of particular areas^47^. Some of the data and models utilized in the current GEP biophysical calculations are flawed, mostly as a result of the frequency and resolution limits of the relevant ecological-environmental monitoring system. Currently, the GEP framework is intended to account for the value of ecosystem fluxes, whereas accounting for the value of ecosystem stocks requires additional study. With additional case studies and technological advancements, however, the GEP calculation will more accurately reflect the value of ecosystem services in the relevant regions.

## Conclusions

Ecosystem services are crucial to the sustainable growth of dense metropolitan environments. Improvements and declines in a region's ecosystems have a direct impact on their capacity to deliver the and services essential to human well-being. The analysis of the BTH megacity demonstrates that GEP can reflect the contribution of a region's ecosystems to human welfare and economic growth. From 2000 to 2020, the GEP of the BTH region exhibited a consistent increasing trend, and the rapid urbanization process in the BTH provided high cultural and product supply values while producing land modifications and a decline in ecosystem regulatory functions. As urbanization progresses, land resources become increasingly scarce and environmental degradation becomes more severe. In the past, the concept of widespread development that promoted economic progress at the expense of the environment was unsustainable. The relationship between urban growth and ecological environment conservation should be balanced to promote the sustainable development of urban agglomerations in accordance with their high-quality development objectives.

### Supplementary Information

Supplementary Information.

## Data Availability

The datasets used or analysed during the current study are available from the corresponding author on reasonable request.
