# Supplementary material for: The impact of China's urbanization on ecosystem service value from the perspective of gross ecosystem product: a case study of Beijing-Tianjin-Hebei region
Source: Sci Rep. 2024 Jul 10;14:15954. doi: 10.1038/s41598-024-64655-8 (PMC11237103; doi:10.1038/s41598-024-64655-8)
Supplement: Supplementary file 1 — Supplementary Information. [file 41598_2024_64655_MOESM1_ESM.docx]

**Section S1 GEP accounting method**

| ***Types of service*** | ***Category of ecosystem services*** | ***Biophysical value accounting methods*** | ***Monetary value accounting methods*** |
| --- | --- | --- | --- |
| ***Material services*** | *Production of forestry,agricultural, animal husbandry, fishery goods，and water supply* | $\text{E}_{\text{pro}}\text{=}\sum_{\text{i}\text{=1}}^{\text{n}} \text{E}_{\text{i}}$  *Where Epro is the total yield ofecosystem products (t/a), Ei is the yield of the ith product (t/a), n is the products category.*  *The annual yield for agricultural products, animal husbandry products, fishery products, forest products, and water usage are reported in forestry, agriculture, fishery and statistics departments.* | ***Market value method***  $\text{V}_{\text{m}}\text{=}\sum_{\text{i}\text{=1}}^{\text{n}} \text{E}_{\text{i}}\text{×}\text{P}_{\text{i}}$  *where Vm is the monetary value of material services (CNY), Pi is the price of category i ecosystem products*  *(CNY / t).* |
| ***Regulating Services*** | *Water retention* | ***Water balance equation***  $\text{Q}_{\text{Wr}}\text{=}\sum_{\text{i}\text{=1}}^{\text{n}} \text{A}_{\text{i}}\text{×}\left（ \text{P}_{\text{i}}\text{-}\text{R}_{\text{i}}\text{-E}\text{T}_{\text{i}} \right）\text{×}\text{10}^{\text{-3}}$  *Where Qwr represents the water retention capacity(m^3^·y^−1^), Pi is the average annual rainfall (mm), Ri is the surface runoff (mm), ETi is the evapotranspira- tion (mm), Ai is the area of i-type ecosystem (m^2^), i is the type of ecosystem in the study area, n is the total number of ecosystem types in the study area .* | ***Shadow engineering method***  *Simulate the construction of water conservancy facilities with the same water storage capacity as the corresponding ecosystems.*  $\text{V}_{\text{wr}}\text{ =}\text{ Q}_{\text{Wr}}\text{×}\text{ C}_{\text{we}}$  *Where Vw represents the accounting value of water retention (CNY / year), c_we_ is the average cost of reservoir construction. The construction cost of reservoirs per unit capacity is firstly used as 8.0 CNY / m^3^ in China in 2017 as the calculation basis.* |
|  | *Soil retention* | ***Universal soil loss equation (USLE)***  ***Qsr* = *R × K × LS ×* (1 *− C × P*)**  *Where Q_sr_ represents the soil retention capacity (t ha^−1^ y^−1^), R is the rainfall erosivity factor, K is the soil erodibility factor, LS is the topographic factor repres- enting the effect of the length of slope, C is the vegetation cover factor, and P is the practice factors of soil erosion control (e.g. terraced fields)* *.* | ***Replacement cost method***  ***Vsr* = *Vsd* + *Vdpd***  *Where V_sr_ represents the accounting val-ue of soil retention (CNY/year), V_sd_ is the reduced cost of dredging (Yuan/year),V_dpd_ is the reduced cost of nonpoint source po-llution treatment (CNY/year).*  ***Vsd* = *λ ×* (*Qsr/ρ*) *× c***  *Where λ is the sediment deposition coefficient , ρ is the soil bulk density (t/m3), c is the cost of reservoir dredg-ing per unit (CNY/m^3^).The cost of reservoir dredging is 17.63 CNY / m^3^.*  ***V_dpd_* = *Qsr × ci × pi***  *Where ci is the content of N and P in sediment, pi is the cost to treat waste water of nitrogen and phosphorus (CNY/t) .The environmental degradation costs of N and P are 875 CNY / t and 2,800 CNY / t.* |
|  | *Flood mitigation* | ***C_fm_= C_rc_ + C_lc_***  *Where Cfm is the total storage of flood water (m^3^), Crc is reservoir and pond flood regulation and storage volume, Clc is the storage of flood water by lakes (m^3^)* *.*  ***C_rc_ = 0.35 × C_t_***  *Where 𝐶_𝑟𝑐_ is the reservoir capacity for flood control (m^3^); C_t_ is the total reservoir capacity (m^3^).*  ***C_lc_ = e^5.808^ × A^0.866^ × 0.98***  *Where A is the lake area .* | ***Shadow project method***  ***V_fm_ = C_fm_ × C_we_***  *Where V_fm_ represents the accounting value of flood mitigation (CNY/year), Cwe is the average cost of reservoir construction. The construction cost of reservoirs per unit capacity is firstly used as 8.0 CNY / m^3^ in China in 2017 as the calculation basis.* |
|  | *Air purification* | $\text{C}_{\text{ap}}\boldsymbol{=}\sum_{\text{i=1}}^{\text{m}} \sum_{\text{j=1}}^{\text{n}} {\text{ }\text{Q}}_{\text{ij}}\text{×}\text{ }\text{A}_{\text{i}}$  *Where C_ap_ is the ecosystem air purification capacity (kg a^−1^), Qij is the purification amount per unit area of the jth air pollutant in the i-type ecosystem (kg km^−2^ a^−1^), A_i_ is the area of type i ecosystem (km^2^) .* | ***Replacement cost method***  *We used the treatment cost of reducing air pollution to calculate the accounting value of air purification from vegetation.*  $\text{V}_{\text{ap}}\text{=}\sum_{\text{i}\text{=1}}^{\text{m}} \sum_{\text{j=1}}^{\text{n}} {\text{ }\text{C}}_{\text{ij}}\text{×}{\text{ }\text{C}}_{\text{j}}$  *Where Cij is the purification amount of the jth air pollutant in the i-type ecosystem (kg a^−1^), cj is the cost of treating j-type air pollutants . SO_2_, NOx, dust treatment cost is 630 CNY / t, 630 CNY / t, 150 CNY / t.* |

| ***Types of service*** | ***Category of ecosystem services*** | ***Biophysical value accounting methods*** | ***Monetary value accounting methods*** |
| --- | --- | --- | --- |
| ***Regulating Services*** | *Water purification* | $\text{C}_{\text{wp}}\text{=}\sum_{\text{i}\text{=}\text{i}}^{\text{n}} \text{Q}_{\text{i}\text{ }}\text{×}\text{A}$  *Where C_wp_ represents the ecosystem water purification capacity (kg a^−1^), Qi is the purification amount per unit area of type i water pollutants, i is the pollutant category, A is wetland area (km^2^).* | ***Replacement cost method***  *We used water treatment costs for removing COD, ammonia nitrogen and total phosphorus to assess the accounting value of the water purification service.*  $\text{V}_{\text{wp}}\text{=}\sum_{\text{i}\text{=}\text{i}}^{\text{n}} \text{Q}_{\text{i}}\text{×}\text{C}_{\text{i}}$  *Where ci is the unit treatment cost of type i water pollutant (CNY/t) .*  *Purification cost of COD, ammonia nitrogen and total phosphorus is 700 CNY/t, 875 CNY/t, 2800 CNY/t.* |
|  | *Carbon sequestration* | ***QtCO_2_=MCO_2_ /Mc×（FCS + GSCS + WCS）***  *Where QtCO_2_ is the total fixed amount of carbon dioxide in terrestrial ecosystem（tCO_2_/a）,FCS is the carbon sequestration of forests (and shrubs)（tC/a）,GSCS is the grassland carbon sequestration（tC/a）,WCS is the carbon sequestration in wetlands（tC/a）,MCO_2_/Mc =44/12 is the coefficient for C to CO_2_.*  ***FCS = FCS × SF × (1+β)***  *Where FCSR is the carbon fixation rate of forest and cluster (tC·ha^-1^·a^-1^), and SF is the forest and cluster area (ha), which is the carbon fixation coefficient of forest and shrub soil.*  *Since the grassland vegetation dries up every year, its fixed carbon is returned back to the atmosphere or enters. In the soil, the carbon fixation amount of the grassland vegetation is not considered, and only the soil carbon fixation amount of the grassland is considered.*  ***GSCS = GSR × SG***  *Where GSR is the carbon fixation rate of the grassland soil (tC·ha^-1^·a^-1^), and SG is the grassland area (ha).*  $\text{WCS=}\sum_{\text{i}\text{=1}}^{\text{n}} \text{SCSR}_{\text{i}}\text{×}\text{SW}_{\text{i}}\text{×}\text{10}^{\text{-2}}$  *Where SCSRi is the carbon fixation rate (g C·m^-2^·a^-1^), SWi is the area of a wetland (ha), i = 1,2, …, n.* | ***Replacement cost method***  ***V_Cf_ =Q_CO2_ × C_C_***  *Where V_Cf_ is the ecosystem carbon fixation value (yuan / a); Q_CO2_ is the total ecosystem carbon fixation tCO_2_ / a), and C_C_ is the carbon price (yuan / t). The carbon price is the same with that in carbon market. The average price of carbon in the Beijing-Tianjin-Hebei region is 58 CNY / t.* |
|  | *Oxygen supply* | ***Q_𝑜𝑝_ =M_𝑂2_ /M_𝐶𝑂2_ × Q_𝐶𝑂2_***  *Where Q_op_ is the coefficient of the ecosystem oxygen release amount (t oxygen / a); 𝑄_𝐶𝑂2_ =32/44 is the conversion from CO_2_ to O_2_, and CO_2_ is the ecosystem carbon fixation amount (tC/a).* | ***Replacement cost method***  ***V_op_ = Q_op_ × Co***  *Where V_op_ is the ecosystem oxygen release value (CNY / a); Q_op_ is the ecosystem oxygen release amount (t oxygen/a), and C_o_ is the industrial oxygen production price (CNY / t). The industrial oxygen production price is 1291.81 CNY / t.* |
|  | *Climate regulation* | *The climate regulation services provided by the ecosystem include temperature maintenance for vegetation transpiration and temperature and humidity maintenance for water surface evaporation.*  $\text{E}_{\text{tt}}\text{=}\text{E}_{\text{pt}}\text{+}\text{E}_{\text{we}}$  $\text{Ept}\text{=}\sum_{\text{i}\text{=1}}^{\text{n}} \text{EPP}_{\text{i}}\text{×}\text{A}_{\text{i}}\text{×}\text{D}\text{×}\text{10}^{\text{6}}\text{/(3600}\text{×}\text{r)}$  $\text{E}_{\text{we}}\text{=}\text{E}_{\text{w}}\text{×}\text{q}\text{×}\text{10}^{\text{3}}\text{/(3600)+}\text{E}_{\text{w}}\text{×}\text{y}$  *Where E_tt_ is the total energy consumed (kw·h/a) by the ecosystem transpiration and evaporation, E_pt_ is the energy consumed from the ecosystem vegetation transpiration (kw·h/a), and E_we_ is the energy consumed by the evaporation of the wetland ecosystem (kw·h/a);* *EPPi is the heat consumed by the transpiration per unit area in the ith-type ecosystem;; Si is the area of the i-type ecosystem (km^2^); D is the working days of the air-conditioner (Days); r is air conditioning energy efficiency ratio:3.0, no magnitude; i is ecosystem type (forest, cluster, grassland); Ew is evaporation (m^3^) (q is volatile latent heat (j/g); humidifier convert 1m^3^ water into steam (kw·h). The heat absorbed by the transpiration of forest and grassland per unit area are 70.40kJ m^-2^ d-1and 25.60kJ m^-2^ d-1, respectively. The vaporization of 1gram of water consumes 2260J of heat, that is, the value of q is 2260 J/ g.* | ***Replacement cost method***  ***V_tt_ = E_tt_× P_e_***  *V_tt_ is the value of ecosystem climate regulation (yuan/a); Ett is the total energy consumed for ecosystem regulation of temperature or humidity (kw·h/a); and Pe is the local electricity price (yuan/ kw·h). The Pe is 0.5 CNY / kwh.* |
|  | *Pest control* | $\text{Q}_{\text{pc}}\text{=}\text{S}_{\text{fpc}}\text{+}\text{S}_{\text{gpc}}$  *Where Q_pc_ is the occurrence area of ecosystem pests and diseases (km²); S_fpc_. is the occurrence area of forest pests and diseases (km²); S_gpc_ is the occurrence area of grassland pests and diseases (km²).* | ***Protection cost method***  ***V_pc_=V_fpc_+V_gpc_***  *Where V_pc_ is the value of pest control (yuan/a); V_fpc_ is the value of forest pest control (yuan/a); V_gpc_ is the value of grassland pest control (yuan/a).*  ***V_fpc_=S_nf_×C_fpc_***  *Where V_fpc_ is the control value of forest pests and diseases (yuan/a); S_nf_ is the natural forest area where pests and diseases are self-healing (km^2^). ; C_fpc_ is the cost of forest pest control per unit area (yuan/km^2^). The forest pest control cost per unit area is 300 CNY / mu.*  ***V_gpe_=S_g_×C_gpe_***  *V_gpe_ is the control value of grassland pests and diseases (yuan/a); S_g_ is the grassland area where the pests and diseases are self-healing (km^2^); C_gpe_ is the grassland pest control cost per unit area (yuan/km^2^). The grassland pest control cost per unit area is 250 CNY / mu.* |
| ***Ecosystem Cultural Service*** | *Natural scenery tourists and consumption* |  | ***Travel cost method***  *Countries located between 30 degrees and 60 degrees of north and south latitudes: there are many cultural attractions, and the distribution of natural scenery and cultural attractions is more complicated. The proportion of natural scenery tourists and consumption to the total number of tourists and consumption is calculated as 60 %.* |

# GEP accounting method and ecosystem service function quantity pricing reference

Jiang, H., Wu, W., Wang, J., Yang, W., Gao, Y., & Duan, Y., (2021). Mapping global value of terrestrial ecosystem services by countries. *Ecosystem Services*, 52. <https://doi.o> rg/10 .1016/j.ecoser.2021.101361.

Ouyang, Z., Zheng, H., Xiao, Y., Polasky, S., Liu, J., Xu, W., Wang, Q., Zhang, L., Xiao, Y., Rao, E., Jiang, L., Lu, F., Wang, X., Yang, G., Gong, S., Wu, B., Zeng, Y., Yang, W., Daily, G.C., (2016). Improvements in ecosystem services from investments in natural capital. *Science*, 352(6292), 1455–1459. https://doi.org/10.1126/science.aaf2295.

Ouyang, Z., Song, C., Zheng, H., Polasky, S., Xiao, Y., Bateman, I.J., Liu, J., Ruckelshaus, M., Shi, F., Xiao, Y., Xu, W., Zou, Z., Daily, G.C., (2020). Using gross ecosystem product (GEP) to value nature in decision making. *Proceedings of the National Academy of Sciences*, 201911439. https://doi.org/10.1073/pnas.1911439117.

Ouyang, X., Tang, L., Wei, X., & Li, Y., (2021). Spatial interaction between urbanization and ecosystem services in chinese urban agglomerations. *Land Use Policy*, 109(4), 105587. <https://doi.org/10.1016/j.la>ndusepol.2021.105587.

Peng, J., Tian, Lu., Liu, Y., Zhao, M., Hu, Y., Wu, J., (2017). Ecosystem services response to urbanization in metropolitan areas: Thresholds identification. *Science of The Total Environment*, 706–714. <https://doi.org/>1 0.1016/j.scitotenv.2017.06.218.

Sun, X., Crittenden, J.C., Li, F., Lu, Z., Dou, X., (2018). Urban expansion simulation and the spatio-temporal changes of ecosystem services, a case study in Atlanta Metropolitan area, USA. *Sci. Total Environ*. 622, 974–987. https://doi.org/10.1016/j.scitotenv.2017.12.062.

Wang, J., Zhou, W., Pickett, S.T.A., Yu, W., & Li, W., (2019). A multiscale analysis of urbanization effects on ecosystem services supply in an urban megaregion. *Science of The Total Environment*, 662(20), 824-833. https://doi.org/10.1016/j.scitotenv.2019.01.260

Wang, H., Liu, L., Yin, L., Shen, J., Li, S., (2020). Exploring the complex relationships and drivers of ecosystem services across different geomorphological types in the Beijing-Tianjin-Hebei region, China (2000-2018). *Ecological Indicators*, 121, 107116. <https://doi.org/10.1016/j.ecol> ind.2020.107116.

Yan, F., Zhang, S., (2019). Ecosystem service decline in response to wetland loss in the Sanjiang Plain, Northeast China. *Ecological Engineering*, 130, 117–121. <https://doi.org/10.1016> /j.ecoleng.2019.02.009.

Zou, Z., Wu, T., Xiao, Y., Song, C., & Ouyang, Z., (2020). Valuing natural capital amidst rapid urbanization: assessing the gross ecosystem product (gep) of china's "chang-zhu-tan" megacity. *Environmental Research Letters*, 15(12). <https://doi.org/10.1088/174> 8-9326/abc2f8.
